# Carbon Dot-Mediated Capillary Electrophoresis Separations of Metallated and Demetallated Forms of Transferrin Protein

**DOI:** 10.3390/molecules24101916

**Published:** 2019-05-18

**Authors:** Leona R. Sirkisoon, Honest C. Makamba, Shingo Saito, Christa L. Colyer

**Affiliations:** 1 Department of Chemistry, Wake Forest University, Winston-Salem, NC 27109, USA; sirklr12@wfu.edu; 2 Razzberry Inc., 5 Science Park, Unit 2E9, New Haven, CT 06511, USA; honest737@gmail.com; 3 Graduate School of Science and Engineering, Saitama University, Saitama 338-8570, Japan; shingo@mail.saitama-u.ac.jp

**Keywords:** carbon dots, capillary electrophoresis, transferrin, metalloproteins, fluorescence

## Abstract

Carbon dots (CDs) are fluorescent nanomaterials used extensively in bioimaging, biosensing and biomedicine. This is due in large part to their biocompatibility, photostability, lower toxicity, and lower cost, compared to inorganic quantum dots or organic dyes. However, little is known about the utility of CDs as separation adjuvants in capillary electrophoresis (CE) separations. CDs were synthesized in-house according to a ‘bottom-up’ method from citric acid or other simple carbon precursors. To demonstrate the applicability of CDs as separation adjuvants, mixtures of holo- (metallated) and apo- (demetallated) forms of transferrin (Tf, an iron transport protein) were analyzed. In the absence of CDs, the proteins were not resolved by a simple CE method; however, upon addition of CDs to the separation buffer, multiple forms of Tf were resolved indicating that CDs are valuable tools to facilitate the separation of analytes by CE. CE parameters including sample preparation, buffer identity, ionic strength, pH, capillary inside diameter, and temperature were optimized. The results suggest that dots synthesized from citric acid provide the best resolution of various different forms of Tf and that CDs are versatile and promising tools to improve current electrophoretic separation methods, especially for metalloprotein analysis.

## 1. Introduction

Carbon dots (CDs) are a unique type of fluorescent nanomaterial consisting of a graphene core decorated with oxygenated functional groups on the surface [1,2,3,4,5]. They are structures comprising of one to a few layers of graphene sheets smaller than 10 nm in diameter. The distinctive photoluminescence of CDs is attributed to the sp^2^ hybridized carbon atoms and the quantum confinement and edge effects resulting from the small size of these carbon-based materials [6]. For example, typical CDs synthesized from citric acid exhibit an emission maximum at 460 nm, independent of excitation wavelength from 300–420 nm, with carboxylic acid and hydroxyl functional groups on the surface [1,6]. CDs interact with potential analytes through hydrophobic, π-π stacking, hydrogen bonding, cation‒π, and electrostatic interactions. The dispersibility of CDs in aqueous solutions is due to the hydroxyl and carbonyl functional groups on their surface, which can be easily altered to render the materials hydrophobic or amphiphilic [2]. CDs exhibit characteristic chemical and physical properties such as biocompatibility, photostability, and low toxicity, and they have the added advantages of simple and low cost synthesis methods. These features have triggered interest in the use of CDs as alternative fluorescence probes in place of organic dyes and inorganic nanoparticles [1,3,5,6,7]. Many recent applications involving CDs capitalize on their fluorescent properties for bioimaging [8,9,10,11], biomedicine [12], and biosensing [13,14,15] to aid in the diagnosis and treatments of diseases, defects, and cancers [1]. However, little is known about the utility of CDs as separation adjuvants in capillary electrophoresis (CE) [3] in comparison to other nanomaterials such as silica nanoparticles [16,17], carbon nanotubes [18], graphene nanoparticles [19], single-walled carbon nanotubes [20], and gold nanoparticles [21,22,23], which have all been reported to enhance CE separations.

CE is a high resolution separation technique that separates analytes based on differential migration rates of charged species in an electric field [24,25]. Advantages of CE include relatively fast analysis times, high efficiency separations, and small sample volumes [3,26]. Further selectivity may be achieved in CE by employing pseudo-stationary phases (solution-based additives present in the separation buffer, which effect the separation of analytes based on their differential associations). The use of pseudo-stationary phases rather than true stationary phases in CE-based methods reduces problems with irreproducibility between capillaries and furthermore, it is simpler than introducing selectivity via the more time-consuming process of immobilization of nanomaterials to form inner capillary wall coatings [27,28]. While surfactants are among the most commonly encountered buffer additives in CE, the use of soluble nanomaterials as buffer additives (or “separation adjuvants”) provides another option for CE method development. For example, Sun and colleagues [3] successfully employed CDs as additives for the separation of cinnamic acid and its derivatives by CE coupled with UV detection and observed increased resolution between cinnamic acid and its derivatives, concluding that CDs are a promising separation material for analytical methods. While carbon nanotubes have be used to assist in protein separations by CE [29], there are no published reports of CDs being used in this capacity and thus, the potential for new developments in this area remains great. Based on these (limited) precedents, we have sought to advance our understanding not only of the versatility and utility of CDs as CE separation adjuvants but also, of metallated protein separations by CE.

In particular, this work focuses on the separation of transferrin (Tf) protein. Tf is a globular, iron transport glycoprotein (comprised of 679 amino acid residues with a molecular weight of 80 kDa). It has two lobes (the N and C lobes) with a high affinity Fe^3+^ binding domain in each [30,31]. When iron is bound to both lobes in Tf (constituting the fully metallated or “holo-” form of the protein), the protein adopts a structural conformation that is more closed (folded) than that of the demetallated (“apo-”) Tf protein. There are four possible conformations of Tf, depending on the number and position of bound Fe^3+^ ions: (i) holo-Tf (fully metallated), (ii) single Fe^3+^ bound only to the C-lobe or (iii) only to the N-lobe (partially metallated), and (iv) apo-Tf (demetallated). The Tf receptor is overexpressed on proliferating cancer cells, but not normal cells; therefore, Tf is a promising carrier protein for targeted drug delivery and therapy for cancerous cells [31,32,33,34,35,36,37,38,39]. The ability to separate the different conformations of Tf (fully metallated, partially metallated, and demetallated), is important because potential drug molecules may have different affinities for the different conformations of Tf. However, a major challenge in separating apo- and holo-Tf by CE is the fact that bound metal ions exert only subtle changes in overall protein mass and charge [40]. This challenge may be met by the use of pseudostationary phases or buffer additives, as demonstrated previously by Nowak and colleagues [26,40], who developed and optimized a CE method for the separation of different forms of Tf using micellar electrokinetic chromatography. Their work, employed sodium dodecyl sulfate and 20% methanol as separation buffer additives, leading to the resolution of apo-Tf, holo-Tf, two partially metallated forms of Tf, lactoferrin, and human serum albumin proteins.

Just as Nowak’s use of surfactants in CE was able to afford greater resolution of metallated and demetallated protein forms, we hypothesized that the use of CDs in CE should likewise afford the necessary selectivity for Tf separations. To this end, CDs were synthesized in-house by pyrolysis of citric acid and other organic precursors. Fluorescence studies were performed to assess the interaction between CDs and apo- and holo-Tf. A significant quenching was observed for the mixture of CDs with holo-Tf and no change in fluorescence signal was observed for CDs with apo-Tf, suggesting that the extent of protein metallation has an impact on protein interaction with CDs. A mixture of holo- and apo-Tf was analyzed by a simple CE method. In the absence of CDs, the proteins were not resolved; however, upon addition of CDs to the separation buffer, multiple forms of Tf were resolved. Sample preparation, buffer identity, ionic strength, pH, capillary inside diameter, and temperature were optimized. The results indicate that dots synthesized from citric acid provide the best resolution between the different metallated forms of Tf. Results from this work indicate that CDs are inexpensive, stable, and convenient buffer additives able to improve current electrophoretic separations of metalloproteins, with implications for greater selectivity in the CE separations of other classes of analyte.

## 2. Results and Discussion

### 2.1. Probing Interactions Between CDs and Tf by Fluorimetry

**Figure 1:**
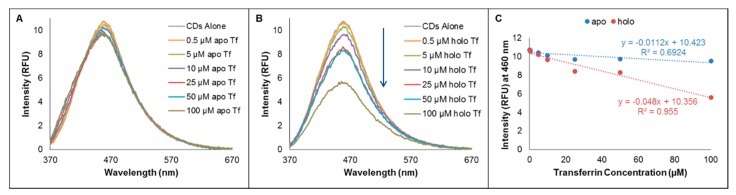
Fluorescence emission spectra for 35 µg/mL samples of oven-synthesized citric acid CDs, with increasing concentrations (from 0.5 µM to 100 µM) of added apo-Tf (**A**) and holo-Tf (**B**). Fluorescence response in terms of intensity at the wavelength of maximum emission (460 nm) as a function of Tf concentration, for apo- and holo-Tf are shown in (**C**). All samples were prepared to the concentrations indicated in the Figures using 50 mM tris-200 mM tricine (pH 7.4) buffer as diluent. The data were corrected for the respective native Tf fluorescence at each concentration. The excitation wavelength was 360 nm and the emission scan range was 365–700 nm.

The interactions between CDs and metallated versus demetallated forms of Tf were assessed by fluorimetry. CDs used in these studies were synthesized by oven pyrolysis of dry citric acid reagent followed by suspension of the resulting CDs in aqueous solution. Fluorescence emission of the CDs alone was measured, followed by emission of the CDs upon addition of increasing amounts of apo-Tf or holo-Tf, as seen in Figure 1. No significant change (11.2% quenching) in fluorescence emission (at 460 nm) was observed for a 35 µg/mL CD sample as the concentration of apo-Tf was increased from 0 to 100 μM (Figure 1A). However, the fluorescence signal was quenched by as much as 47.6% upon the addition of up to 100 μM holo-Tf to the same CD sample (see Figure 1B). The intensities represented in Figure 1C were determined at the wavelength of maximum fluorescence emission (460 nm) after having corrected for native Tf fluorescence at each concentration (as shown in Figure S1-A,B) and applying a five-point boxcar smoothing. The extent of change in fluorescence of CDs as a function of Tf protein concentration is represented by the slopes of the response curves in Figure 1C. The slope for apo-Tf is −0.0112 RFU/µM indicating very little to no change in fluorescence of the CDs. However, the slope for holo-Tf is −0.048 RFU/µM, revealing a direct proportionality between the extent of fluorescence quenching of the CDs signal and the concentration of holo-Tf. In work by Bhattacharya and colleagues [39] a similar effect was characterized as static quenching via their steady-state and time-resolved photoluminescence measurements at pH 7.4. Based on estimated thermodynamic parameters of the CD-Tf association determined from quenching measurements performed at various temperatures, they concluded that the observed quenching was a result of the electrostatic interaction between CDs and the Fe^3+^ ions associated with holo-Tf, not the amino acid residues. Furthermore, Zhu and coworkers [41] showed that the presence of Fe^3+^ ions in bulk solution quenched the intrinsic fluorescence of CDs. Therefore, we believe the differential effect of apo- versus holo-Tf on the fluorescence of CDs in our experiments is most likely a result of the paramagnetic property of the Fe^3+^ ions of the holo-Tf impacting the quantum yield. However, such an effect does not preclude the possibility of different metallated protein states interacting to different extents with the CDs (and we explore this possibility in more detail in the capillary electrophoresis studies discussed in Section 2.2).

Additionally, the experiment was repeated using CDs synthesized in the autoclave and suspended in aqueous solution. A similar trend was observed with these CDs: little to no change in fluorescence emission of the CDs upon increasing the concentration of apo-Tf (Figure S-1D), and decreased fluorescence emission upon increasing the concentration of holo-Tf (Figure S-1E). The conformation of demetallated apo-Tf is such that it has two tryrosine, one aspartate, and one histidine residue exposed [39]. While it seems plausible that these exposed residues could interact with the CDs (via hydrophobic, π-π stacking, H-bonding, or electrostatic interactions), the relative lack of change in fluorescence emission of apo-Tf with CDs could not provide evidence for any such interactions under the solution conditions employed here. However, the observed fluorescence quenching of CDs with holo-Tf indicates that the bound Fe^3+^ in the metallated form of the protein experiences electrostatic interactions with the hydroxyl and carboxylic acid groups on the surface of the CDs, resulting in a non-emissive ground state complex [39]. Thus, even though CDs may interact (to a different extent) with demetallated and metallated forms of Tf, this could not be confirmed by fluorescence studies alone.

### 2.2. CE Method Development and Optimization for the Separation of Apo-Tf and Holo-Tf

#### 2.2.1. Studying the Effects of Sample Preparation: Diluent and Sample Additives

**Figure 2:**
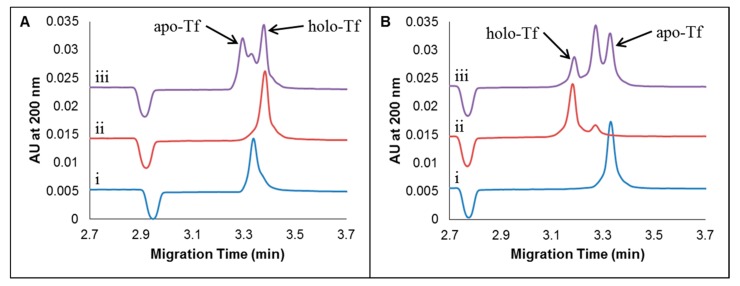
Effects of oven CDs as additives for samples of apo-Tf, holo-Tf and mixtures of apo- and holo-Tf (25 μM each) without CDs (**A**) and with CDs (**B**) for 25 μM apo-Tf (i), 25 μM holo-Tf (ii), and a mixture of apo- and holo-Tf (iii). Electropherograms are vertically offset for clarity. A volume of 1.25 nL (5.2 s at 1.3 psi) was injected and 20 kV was applied. The separation occurred on a Beckman Coulter P/ACE MDQ System coupled with a UV detector at 15 °C on a 25 μm i.d. capillary with an effective length of 30 cm and a total length of 40 cm.

Given the differential interactions of CDs with metallated versus demetallated forms of Tf, as evidenced by differences in fluorescence quenching (Section 2.1), we surmised that CDs might be useful in the separation of these protein forms. Samples of apo-Tf, holo-Tf, and mixtures of apo- and holo-Tf were first prepared in aqueous solution alone and then subjected to analysis by CE with UV absorbance detection, employing a 50 mM tris-200 mM tricine (pH 7.4) separation buffer. Typical electropherograms resulting from these protein samples prepared in aqueous solution–with no CDs–are shown in Figure 2A. Subsequently, the water-based Tf samples and the separation buffer were prepared with added CDs (such that the final concentration of dots was 35 µg/mL in all cases), and the resulting electropherograms are shown in Figure 2B. The CDs used for these CE experiments were synthesized from citric acid by oven pyrolysis, followed by suspension in 50 mM NaOH and dialysis against ultrapure water for eight hours prior to use, unless otherwise stated.

The blue traces (i) in Figure 2A,B represent apo-Tf samples without and with added CDs, respectively. While there was no significant change in the observed migration time of the apo-Tf peak as a result of adding CDs to the sample (and separation buffer), there was a marked change (50.6%) in the (negative) electrophoretic mobility μ_ep_ of apo-Tf (from −0.00239 cm^2^ V^−1^ s^−1^ in the absence of CDs to −0.00360 cm^2^ V^−1^ s^−1^ in the presence of CDs). This change in (negative) electrophoretic mobility of apo-Tf was accompanied by a 34.0% increase in peak height and a 44.4% increase in peak area. The increase in (negative) electrophoretic mobility may provide evidence of the association of apo-Tf with CDs to produce a larger complex with greater net negative charge. Such a complex with greater negative electrophoretic mobility would move counter to the direction of electroosmotic flow, and so might be expected to appear at a longer migration time in the resulting electropherogram. However, based on the position of the small negative marker peak in Figure 2A,B, the electroosmotic mobility was found to increase by 5.4% (from 0.0205 cm^2^ V^−1^ s^−1^ to 0.0216 cm^2^ V^−1^ s^−1^) upon the addition of CDs to the buffer system. In this particular case, the combination of the increased electroosmotic mobility and the decreased (i.e., increased negative) electrophoretic mobility resulted in very little change in the net mobility of apo-Tf (with and without added CDs) and thus the migration time of the apo-Tf peak appeared virtually unchanged. The increase in apo-Tf peak height and area in the system containing CDs may provide further evidence of the formation of apo-Tf‒CD complexes, since such complexes may demonstrate some variation in size and enhanced absorbance relative to free apo-Tf.

The red traces (ii) in Figure 2A,B represent holo-Tf samples without and with added CDs, respectively. A 6.0% decrease in migration time of holo-Tf (from 3.38 min to 3.18 min) was observed upon the addition of CDs. This reduced migration time is due to an increase in net mobility, and recall that net mobility is given by the sum of electroosmotic and electrophoretic mobilities. In the case of holo-Tf, it appears that the impact of added CDs on the electroosmotic flow (recall, a 5.9% increase in electroosmotic mobility was observed) was greater than the impact of added CDs on the electrophoretic mobility of the protein. The electrophoretic mobility of holo-Tf was found to be −0.00281 cm^2^ V^−1^ s^−1^ in the absence of CDs and −0.00278 cm^2^ V^−1^ s^−1^ in the presence of CDs, which represents just a 1.1% decrease (in the negative electrophoretic mobility, which is effectively the same as a 1.1% increase in μ_ep_ towards the cathode). This change is small in comparison to the 50.6% change in electrophoretic mobility observed for apo-Tf, which might suggest that the demetallated form of the protein has a greater affinity for (or forms more stable, long-lived complexes with) CDs compared to the metallated form of the protein. Thus, in the case of holo-Tf, the relatively small change in electrophoretic mobility is overshadowed by a greater change in electroosmotic flow upon the addition of CDs to the sample and separation buffers, which translates into a greater net mobility and shorter migration time.

The peak height of the primary holo-Tf peak decreased 19.1% and the area increased by 15.5% upon the addition of CDs (Figure 2A(ii) vs. Figure 2B(ii)). The decrease in peak height and increase in peak area is attributed to the loss of Fe^3+^ ions by holo-Tf [40] while the appearance of a new, smaller peak at 3.25 min (see Figure 2B(ii)) is attributed to a partially metallated form of Tf, which may associate with CDs in the separation buffer to a different extent than does the fully metallated form of Tf from which it originates. This appearance of an additional peak induced by the addition of CDs to the holo-Tf sample, taken together with changes in migration times or net mobilities, supports the idea of differential interactions between CDs with various different metallated forms of Tf.

Whereas samples of individual Tf proteins in the absence of CDs gave rise to single peaks (Figure 2A(i and ii)), a sample mixture containing 25 μM each of apo- and holo-Tf in water (also in the absence of CDs) gave rise to an unresolved cluster of three peaks by CE (Figure 2A(iii)). In the protein mixture, there is presumably an opportunity for exchange of Fe^3+^ ions between protein forms, resulting in unresolved metallated, demetallated, and partially metallated Tf proteins. Upon the addition of CDs, the cluster of three peaks was more clearly resolved in the electropherogram for the mixed-protein sample (Figure 2B(iii)). Interestingly, the combined area of the mixture increased 22.6%, and the migration order of apo-Tf and holo-Tf was reversed in the electropherogram of the protein mixture upon the addition of CDs to the sample and separation buffer. Whereas holo-Tf migrated last in the sample containing a mixture of proteins in the absence of CDs, it migrated first in the sample containing CDs. As discussed previously, this change in the proteins’ net mobilities, brought about by the addition of CDs to the buffer system, may be attributed to the combined effects of a change in electroosmotic mobility and a change in electrophoretic mobility due to associations between CDs and Tf proteins. The overall impact was improved resolution of the protein mixture.

To further ascertain the importance of sample composition on CE resolution, Tf samples were prepared using the separation buffer (50 mM tris-200 mM tricine, pH 7.4) as a diluent rather than using pure water, without or with added CDs (35 µg/mL). Representative electropherograms are shown in Figure S2-A,B, respectively. Additionally, Tf samples were prepared in the buffer of 25 mM tris-100 mM tricine (pH 7.4). Representative electropherograms for these Tf samples without added CDs and with 17.5 μg/mL added CDs are shown in Figure S2-C,D, respectively. No significant improvement (nor deterioration) in separation efficiency was afforded by the changes sample buffer concentrations studied.

A comparison of Figure 2 and Figure S-2 leads us to conclude that an enhancement of the CE separation of apo- and holo-Tf is achieved in the presence of CDs regardless of sample composition. That is, preparations of Tf samples in water, separation buffer, and diluted separation buffer all resulted in similar electropherograms. The electropherograms for mixed samples containing both apo-Tf and holo-Tf protein standards revealed the appearance of a third peak, which was better resolved upon the addition of CDs to the sample and separation buffer. The appearance of this third peak upon mixing apo-Tf and holo-Tf together may indicate a partial exchange of Fe^3+^ from the holo-Tf to apo-Tf when mixed. Intraconversion between metallated and demetallated forms of Tf has been documented elsewhere [40]. In all cases, resolution improved upon the addition of CDs. In Figure 2, for example, the peak attributed to a partially metallated Tf species is better resolved from the apo-Tf peak (R_s_ = 0.5 without CDs and R_s_ = 1.1 with CDs) and it is also better resolved from the holo-Tf peak (R_s_ = 0.8 without CDs and R_s_ = 1.5 with CDs) in mixed protein samples. This suggests that the CDs interact differentially with each form of Tf, presumably due to differing contributions from hydrophobic, π-π stacking, H-bonding, or electrostatic interactions in the absence and presence of metal in various folded states of the proteins. Regardless of the sample preparation (that is, protein in water, 25 mM tris-100 mM tricine, or 50 mM tris-200 mM tricine), apo-Tf migrated first and holo-Tf last in the absence of CDs; however, in the presence of CDs, holo-Tf migrated first and apo-Tf last. Furthermore, since the effect of sample buffer ions on the resolution of a mixture of Tf protein forms was nominal relative to the effect of added CDs, method development is not constrained to a single sample preparation, giving the analyst greater flexibility when optimizing metalloprotein separations by this CD-enhanced CE method. Based on simplicity, ultrapure water with added CDs was chosen for Tf sample preparations in subsequent studies.

Whereas CDs were introduced simultaneously to both the sample preparation and the separation buffer to improve the separation of mixtures of apo-Tf and holo-Tf, as described above, the impact of CDs as separation adjuvants for on-column use only (CDs only in the separation buffer) and pre-column use only (CDs only in the sample preparation) was also explored. Pre-column use of CDs (as additives to the sample preparation only) did not result in a significant improvement in resolution of Tf protein forms (Figure S-3ii) relative to the use of no added CDs (Figure S-3i). However, CDs added to the separation buffer alone led to improved resolution of a mixture of Tf proteins relative to separations conducted without added CDs, as seen in Figures S-3-iii and S-3-iv relative to S-3-i. The resolution achieved with CDs in the separation buffer alone was still not as good as the resolution achieved with CDs in both the sample buffer and the separation buffer (Figure S-3-iv, and previously, Figure 2B-iii), and so CDs were employed as additives to both sample and separation buffers in all following CE experiments.

The effects of changes to CD composition on the resolution of the three Tf peaks observed for a mixture of apo- and holo-Tf with CDs were tested. Carbon dot composition was altered by replacing citric acid as the organic precursor with ascorbic acid, gluconic acid, N-acetylneuraminic acid, or glucose. Figure S-4 shows representative electropherograms employing these altered CDs (35 µg/mL) as adjuvants in the separation of mixtures of apo- and holo-Tf with UV detection at 200 nm (Figure S-4A) and with LIF detection using a 375 nm laser and a 400 nm long pass filter (Figure S-4B). Altering dot composition by using different precursors resulted in some CDs with the potential to improve the resolution of the individual components in a mixture of apo- and holo-Tf with further optimization and others that did not affect the mobility at all as observed by UV detection and a single peak or broad hump from LIF detection. Although all of the chosen precursors result in CDs decorated with hydroxyl and carboxylic acid functional groups, the differences in their interaction with apo- and holo-Tf could be due to the ratio of carboxylic acid to hydroxyl functional groups on the surface, or differences in the carbon dot core, which may result from the arrangement of each precursor molecule as the carbon dot core was built, leading to the differences in the intrinsic fluorescence of the CDs from each precursor. Overall, CDs prepared from citric acid yielded the best resolution between the metallated, partially metallated, and demetallated forms of Tf.

Incubation time studies ranging from 2–197 min (time elapsed between preparation of Tf protein samples with added CDs and their analysis by CE) revealed no correlation between sample incubation time and peak area or migration time (data not shown). Thus, CDs can be employed as separation adjuvants in CE studies without imposing any additional restrictions on method or time of sample preparation. This lends further credence to the utility of CDs as CE separation adjuvants.

#### 2.2.2. Effect of Concentration of Added CDs

**Figure 3:**
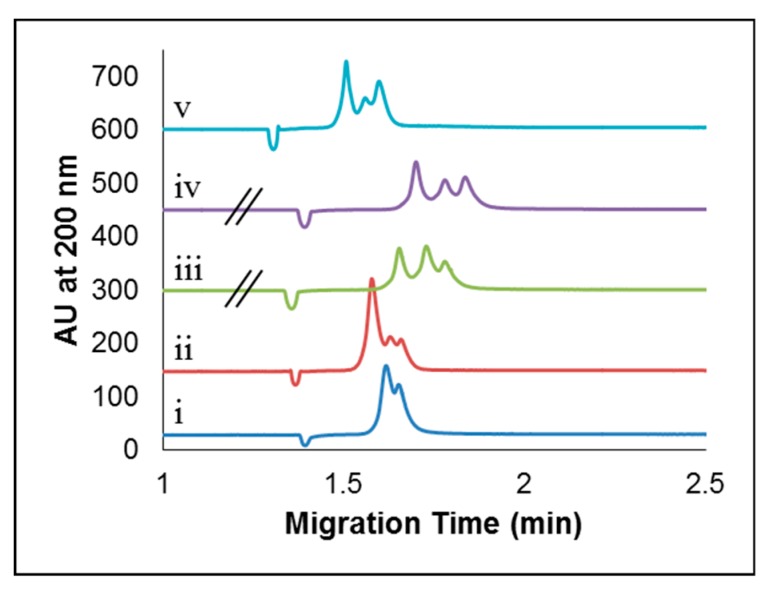
Abbreviated range of CDs concentrations tested with a mixture of apo- and holo-Tf (25 µM each). Concentrations of CDs shown are (**i**) 2 μg/mL, (**ii**) 10 μg/mL, (**iii**) 35 μg/mL, (**iv**) 100 μg/mL, and (**v**) 500 μg/mL. Electropherograms are vertically offset for clarity. A volume of 5 nL (2.1 s at 45 mbar) was injected and 20 kV was applied. The separation occurred on an Agilent G1600A CE coupled with a DAD UV/Vis Detector at 25 °C on a 50 μm i.d. capillary with an effective length of 24 cm and a total length of 32.5 cm.

CE experiments were conducted with different concentrations of CDs added to the sample preparations and separation buffer in order to determine the optimal concentration to enhance the separation of a mixture of apo- and holo-Tf. The concentrations of CDs tested were 2, 5, 7, 10, 25, 35, 50, 75, 100, 250 and 500 µg/mL. A subset of these representative electropherograms are shown in Figure 3 (with the full concentration range shown in Figure S-5). At CD concentrations of 2–7 µg/mL, only two peaks were observed for a sample mixture containing 25 μM each of apo-Tf and holo-Tf (Figure 3-i). The addition of 10 µg/mL CDs gave rise to a broad signal with three unresolved components (Figure 3-ii). Upon the addition of anywhere from 25–500 µg/mL of CDs to the sample and separation buffer, three nearly resolved peaks were observed (Figure 3iii–v). It should be noted that sample compositions in Figure 3 differ from the optimal sample conditions shown in Figure 2 (optimal), due to the sequencing of experiments conducted. The samples in Figure 3 were prepared in 25 mM tris-100 mM tricine buffer (pH 7.4), with a concentration of CDs in the sample equal to half of the concentration in the separation buffer.

Based on the results in Figure 3 (and Figure S-5), we determined the optimal concentration of CDs to be 35 µg/mL for the CE separation of the sample mixture of apo- and holo-Tf. While 25–500 µg/mL CDs also permitted the partial resolution of three peaks (attributed to apo-Tf, holo-Tf, and partially metallated Tf in the sample), the use of 35 µg/mL CDs was chosen as a conservative value to afford the necessary separation while also accommodating any synthetic variations from different batches of CDs, or effects due to post synthesis clean-up, and to prevent a high baseline from the absorbance of the CDs should they have been used at higher concentrations.

#### 2.2.3. Separation Buffer Composition: Background Electrolyte, pH, and Concentration Effects

**Figure 4:**
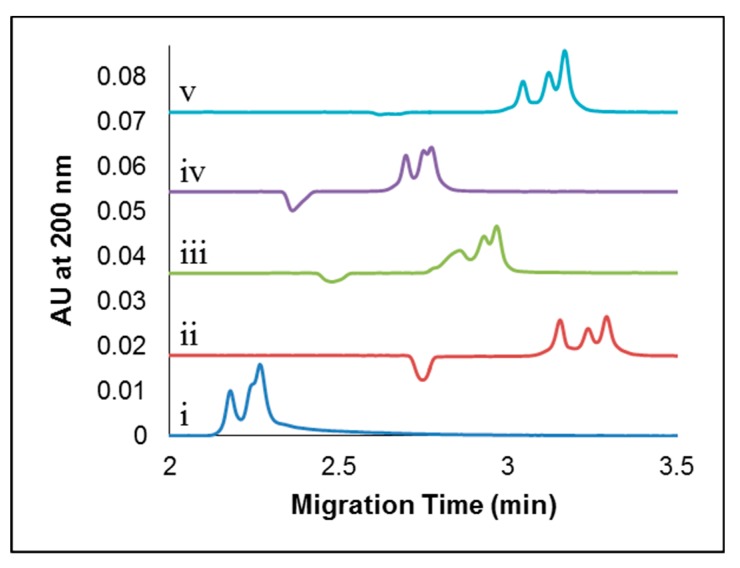
Separation buffer composition study for mixtures of apo- and holo-Tf (25 μM each) with CDs at pH 7.4 in different separation buffers. The separation buffers used were 10 mM phosphate (**i**), 50 mM tris-200 mM tricine (**ii**), 50 mM tris-200 mM glycine (**iii**), 50 mm tris-500 mM glycine (**iv**), and 50 mM tris-HCl (**v**). Electropherograms are vertically offset for clarity. A volume of 1.25 nL (5.2 sec at 1.3 psi) was injected and 20 kV was applied. The separation occurred on a Beckman Coulter P/ACE MDQ System coupled with a UV detector at 25 °C on a 25 μm i.d. capillary with an effective length of 30 cm and a total length of 40 cm.

A variety of different background electrolytes were tested as separation buffers in order to determine their effects on separation efficiency for sample mixtures containing apo- and holo-Tf. These included buffers composed of phosphate, tris-tricine, tris-glycine, and tris-HCl, all at pH 7.4. Representative electropherograms obtained using each of these separation buffers for the analysis of a sample mixture containing 25 μM each of apo-Tf and holo-Tf with 35 μg/mL CDs are shown in Figure 4i–v. Tris-tricine and tris-HCl separation buffers at pH 7.4 (Figure 4-ii and Figure 4-v, respectively) afforded the best resolution (with three nearly resolved peaks representing apo-Tf, holo-Tf, and partially metallated Tf), albeit with the longest migration times relative to the other separation buffers tested. Calculated resolution values are summarized in Table S-2. The remaining separation buffers at pH 7.4 (10 mM phosphate, Figure 4-i; 50 mM tris-200 mM glycine, Figure 4-iii; 50 mM tris-500 mM glycine, Figure 4-iv) yielded faster eluting, unresolved peaks and so were not preferred above the tris-tricine and tris-HCl buffers.

Subsequently, the effect of separation buffer pH on the resolution of a mixture of apo- and holo-Tf with CDs was evaluated with phosphate and tris-tricine separation buffers at pH 4.4, 7.4, and 10.4. No signal was observed in electropherograms recorded at pH 4.4 for both tris-tricine and phosphate buffers. At pH 7.4 the tris-tricine buffer gave rise to three peaks while the phosphate buffer gave rise to only two peaks (Figure S-6-i and S-6-ii, respectively), while at pH 10.4 only one peak was observed for both tris-tricine and phosphate buffers (Figure S-6-iii and S-6-iv, respectively). The lack of resolution afforded by pH 4.4 and 10.4 separation buffers and by phosphate buffer relative to tris-tricine buffer at all pHs tested, led us to conclude that the tris-tricine buffer at pH 7.4 (with CDs) was optimal for the resolution of a sample mixture containing apo-Tf and holo-Tf (also with CDs). However, the optimal concentration for the tris-tricine buffer remained to be determined, and so a concentration study was undertaken, as described presently.

With a fixed concentration of 50.0 mM tris, we varied the concentration of tricine from 100.0–300.0 mM to create a series of separation buffers (each adjusted to pH 7.4, if necessary) in order to determine the optimum buffer concentration for this work. Representative electropherograms recorded for a Tf mixture sample using the various concentrations of tris-tricine separation buffer (at pH 7.4, both with and without CDs) are shown in Figure S-7. At or above tricine concentrations of 200 mM we observed a significant increase in migration time for Tf; however, the increased resolution afforded by these higher concentration buffers, especially in the range of 200–250 mM tricine relative to 100–175 mM tricine, suggested that 200 mM tricine was optimal.

Using this, we subsequently varied the tris concentration from 25.0–100.0 mM in the separation buffer while maintaining a pH of 7.4 to complete the buffer optimization process. Representative electropherograms for a sample mixture of apo- and holo-Tf revealed three peaks for 200 mM tricine separation buffers containing CDs with either 25 mM or 50 mM tris (Figure S-8-i and S-8-ii, respectively), but only two peaks were resolved with the higher concentrations of tris in the separation buffer (75 mM, Figure S-8(iii); 100 mM, Figure S-8(iv)). At all concentrations, we again verified that the presence of CDs (in the tris-tricine buffer and the Tf sample) was essential to achieving resolution of the various metallated forms of the protein (Figure S-8). Hence, 50 mM tris-200 mM tricine (pH 7.4) containing 35 μg/mL CDs was chosen as the optimal separation buffer for this method.

#### 2.2.4. Optimizing Capillary Inside Diameter and Temperature

In addition to optimizing the separation buffer and sample preparation including CDs, we likewise studied the effects of capillary inside diameter and temperature on the resolution of a mixture of apo- and holo-Tf in order to optimize the overall separation method. Separations were conducted using 20, 25, and 50 μm i.d. capillaries (as shown in Figure S-9A, S-9B, and S-9C, respectively), each thermostated at 15, 25, or 30 °C, with the optimized buffer and sample conditions determined herein. Interestingly, variation in capillary inside diameter and temperature within the ranges conducted in this study did not have a marked impact on separation efficiencies. As expected, increased temperatures led to decreased migration times (due to reduced buffer viscosities), and the largest capillary (50 μm i.d.) produced broader, less resolved signals with greater absolute absorbances (due to greater sample loading). Based on these results, the 25 µm i.d. capillary operated at 15°C (Figure S-9Bi) was chosen as optimal for this method.

Thus, the final optimized CE method, designed to afford the greatest resolution of a sample mixture containing various metallated forms of Tf protein, employs a 25 μm i.d. capillary at 15 °C with a 50 mM tris-200 mM tricine separation buffer (pH 7.4) containing 35 µg/mL CDs, and samples prepared or sample buffer with 35 µg/mL CDs added.

## 3. Materials and Methods

### 3.1. Reagents

Citric acid (>99.5%) and glycine (≥99.0%, NT) were purchased from Sigma-Aldrich (St. Louis, MO, USA). Sodium phosphate dibasic (ACS Grade), HCl (ACS Grade), and NaOH (ACS Grade) were all purchased from Fisher Scientific (Suwanee, GA, USA). Human apo-transferrin (“Apo-Tf”) (≥95%) and human holo-Tf (≥95%) were purchased from Calbiochem (San Diego, CA, USA). Tricine (electrophoresis grade) was purchased from MP Biomedicals (Solon, OH, USA) and tris(hydroxymethyl)aminomethane (proteomics grade, Amresco Life Science, Solon, OH, USA) was purchased from VWR (Atlanta, GA, USA). Ultrapure water, purified using a Milli-Q^®^ Reagent Water System from EMD Millipore Corporation (Billerica, MA, USA), was used for all aqueous samples and solutions.

### 3.2. Carbon Dots

The CDs used in this work were prepared in-house following the method from Dong et al. [6] with some modifications. Briefly, 2 g of dry citric acid in a 20-mL disposable scintillation vial was heated in an Isotemp oven (model 506G; Fisher Scientific, Waltham, MA, USA) at 180 °C for four hours. Alternatively, 2 g of dry citric acid was placed in a 50 mL Teflon autoclave liner, which was placed into a standard stainless steel 304 autoclave reactor (purchased from Labware on Amazon.com, part number: 2T50, Wilmington, DE, USA) and heated in the oven at 180 °C for 24 hours. The resulting dark orange liquid was cooled slightly and a 20 mL aqueous solution of 50 mM NaOH was added to the scintillation vial (or autoclave reactor) and was sonicated using a 2510 Branson sonicator (Branson Ultrasonics Corporation, Danbury, CT, USA) for 30 min to suspend the CDs. Three, 0.5-mL aliquots of the resulting neat CD solutions were lyophilized with a FreeZone 2.5 Liter −84 °C Benchtop Freeze Dryer (Labconco, Kansas City, MO, USA), and the mass of the resulting dried product was found. The average mass of three dried CD aliquots was found in order to provide a representative mass-per-volume (mg/mL) concentration of CDs for the batch. In some cases, a post-synthesis cleanup was performed by dialyzing (Float-a-Lyzer G2, MWCO 500-1000 D, from Spectrum Labs, (purchased from Fisher Scientific, Suwanee, GA, USA)) about 5 mL of the neat CD solution against water for eight hours, changing the water every two hours.

### 3.3. Separation Buffer and Sample Preparation

Stock solutions (1.00 M) of each buffer component (tris and tricine) were prepared separately by dissolving the appropriate mass of reagent in water, quantitatively transferring to a volumetric flask and filling to the line with ultrapure water. The resulting stock solutions were filtered (0.2 µm nylon syringe filter, VWR) and stored in a polypropylene or HDPE vessel at 2–8 °C until needed. The stock solutions were brought to room temperature before use. The tris-tricine buffer used for fluorescence emission and CE studies, was prepared to a final concentration of 50.0 mM tris and 200.0 mM tricine (unadjusted pH 7.4).

Additionally, other separation buffers were prepared from phosphate, tris and glycine. The phosphate separation buffer was prepared to a final concentration of 10.0 mM dibasic sodium phosphate adjusted to pH 7.4 with 1.0 M phosphoric acid. Two different tris-glycine buffers were prepared, one with a final concentration of 50.0 mM tris-200.0 mM glycine, and the other with 50.0 mM tris-500.0 mM glycine, and both adjusted to pH 7.4 by the dropwise addition of 1.0 M HCl. Lastly, a tris-HCl separation buffer was prepared to a final concentration of 50.0 mM tris adjusted to pH 7.4 with 1.0 M HCl.

Separate apo- and holo-Tf stock solutions (250 µM each) were prepared by dissolving the 0.01 g of apo-Tf or holo-Tf in the 500 µL of filtered ultrapure water (0.2 µm, nylon syringe filter) in a 1.6 mL microcentrifuge vial. Unused Tf stock solutions (of apo- and holo-Tf, separately) were portioned into 5 µL aliquots and stored at −20 °C until needed.

Samples were prepared for analysis by adding the appropriate volumes of apo-Tf, holo-Tf, or both stock solution(s) to a 1.6 mL microcentrifuge vial (Fisher Scientific, Suwanee, GA, USA) for fluorimetry studies and a 0.6 mL microcentrifuge vial (Fisher Scientific) for CE studies, followed by dilution with the appropriate volume of buffer (50 mM tris-200 mM tricine pH 7.4) for fluorimetry measurements, and with the appropriate volumes of sample buffer (100 mM tris-400 mM tricine pH 7.4, with 70 µg/mL CDs when present) and ultrapure water (producing a sample with a total volume of 50 µL) such that the final buffer concentration was (50 mM tris-200 mM tricine pH 7.4) for CE samples. The final buffer concentrations for fluorimetry samples are shown in Table S-1. For fluorimetry studies, a working solution of CDs (350 µg/mL) was prepared each time the studies were performed, from the neat solution of CDs after sonication of the neat solution for 5 min followed by approximately 25-fold dilution of a 39.78 µL portion of the neat solution with 960.22 µL buffer. The working solution was sonicated for 1–2 min prior to transferring the appropriate volume to the microcentrifuge vial containing the diluted Tf protein immediately prior to analysis (producing a sample with total volume of 500 µL). For CE samples, neat CDs solution (a 25.6 µL aliquot) was diluted approximately 390 fold with separation buffer in a 10.00 mL volumetric flask (resulting in a separation buffer with a final concentration of 35 µg/mL CDs), and a 2.56 µL aliquot of neat CDs solution was diluted 195 fold with 497.44 µL sample buffer in a 1.6 mL microcentrifuge tube (resulting in sample buffer with a final concentration of 70 µg/mL CDs). The sample vial was then vortexed to mix all constituents, and the solution therein (containing various combinations of apo-Tf, holo-Tf, buffer, and CDs, as needed) was transferred to a clean, dry, injection vial (Agilent, 250 µL or Beckman, 200 µL) for CE studies or to a semi-micro quartz cuvette (Fisher Scientific) for fluorimetry studies. The cuvette was cleaned by triple-rinsing with water and with 95% ethanol (Fisher Scientific).

### 3.4. Instrumentation

Spectrofluorimetry studies were conducted using a Cary Eclipse fluorescence spectrophotometer (Agilent Technologies, Foster City, CA, USA). An excitation wavelength of 360 nm was used, followed by an emission scan from 365–700 nm. Excitation and emission slit widths were 5 nm; the scan rate was 300 nm/min; and the PMT voltage was 600 V. CE studies were conducted using a P/ACE MDQ CE System with 32Karat software (Beckman Coulter, Redwood City, CA, USA) or an Agilent G1600A CE System equipped with Chemstation software. Detection was performed by UV absorbance at 200 nm, or by laser-induced fluorescence (LIF) using a 375 nm diode laser with 5 mW output power (Oz Optics Ltd., Carp, ON, Canada) and 400 nm long pass filter (Omega Optical, Brattleboro, VT, USA), or a Picometrics LIF Detector (406 nm laser with 12.5 mW output power and 410 nm emission filter) for the Beckman-Coulter and Agilent CE systems, respectively. All CE experiments employed uncoated fused-silica capillaries (Polymicro Technologies, Phoenix, AZ, USA) with different lengths and inside diameters (as specified in the Results and Discussion section).

## 4. Conclusions

The use of CDs as separation adjuvants in CE method development is presented as an opportunity to expand upon the usual repertoire of pseudo-stationary phases and buffer additives for enhanced separations. CDs employed in this study were synthesized in-house by a simple method of oven pyrolysis of citric acid. It is of significance that CDs were found to interact differentially with the various forms of Tf protein (metallated, demetallated, and partially metallated), as evidenced by varying extents of fluorescence quenching (which occurred for holo-Tf but not apo-Tf), and by a much more pronounced change in electrophoretic mobility for apo-Tf relative to holo-Tf with CDs present in the sample and separation buffers. This differential association of CDs with metallated and demetallated proteins facilitated greater resolution of apo- and holo-Tf by CE, along with the added ability to discern an additional sample component in the resulting electropherograms, which is presumed to be a partially metallated form of the protein, arising from spontaneous metal ion exchange between holo-Tf and apo-Tf components of the sample. Specifically, by employing a 25 μm i.d. capillary at 15 °C with a 50 mM tris-200 mM tricine separation buffer (pH 7.4) containing 35 µg/mL CDs, we were able to resolve three peaks for a sample comprising 25 μM each of apo-Tf and holo-Tf with 35 µg/mL CDs in water. Most importantly, resolution of these sample components was not possible in the absence of CDs. These results indicate that CDs are useful as CE buffer additives and can lead to improved resolution for challenging samples such as metallated protein mixtures. The application of CDs to other separation challenges in CE is a promising avenue for future studies.

Improvements to the method presented herein include efforts to resolve the two partially metallated forms of Tf co-migrating as the middle peak in the separations of mixtures of apo- and holo-Tf with CDs synthesized from citric acid, through modifications of the CDs, such as the addition of nitrogen groups or passivation with polymer, further optimization of the CE separation of mixtures of apo- and holo-Tf involving CDs from *N*-acetylneuraminic acid or glucose, optimization of the separation voltage, and determining if the partially metallated form of Tf is eliminated or reduced by the addition of 20% methanol to the separation buffer, which was found help reduce the loss of Fe^3+^ ions form holo Tf. Additionally, the benefits of CDs in a polymer enhanced capillary transient isotachophoresis (PectI) method for mixtures of apo- and holo-Tf will be investigated.

## Supplementary Materials

The following are available online, Table S-1: Final Buffer Concentrations for Fluorimetry Samples; Table S-2: Resolution Values for Figure 4; Figure S-1: Fluorescence spectra of apo- and holo-Tf without CDs and with Autoclave CDs; Figures S-2–S-9, representative electropherograms for method development and optimization studies.
