# Supplementary material for: Carbon Dot-Mediated Capillary Electrophoresis Separations of Metallated and Demetallated Forms of Transferrin Protein
Source: Molecules. 2019 May 18;24(10):1916. doi: 10.3390/molecules24101916 (PMC6571678; doi:10.3390/molecules24101916)
Supplement: Supplementary file 1 [file molecules-24-01916-s001.pdf]

Supplementary Material

Carbon Dot-Mediated Capillary Electrophoresis Separations of Metallated and Demetallated Forms of Transferrin Protein

Leona R. Sirkisoon [1], Honest C. Makamba [2], Shingo Saito [3], and Christa L. Colyer [1,*]

[1] Department of Chemistry, Wake Forest University, Winston-Salem, NC 27109, USA; sirklr12@wfu.edu
[2] Razzberry Inc., 5 Science Park, Unit 2E9, New Haven, CT 06511, USA; honest737@gmail.com
[3] Graduate School of Science and Engineering, Saitama University, Saitama 338-8570, Japan; shingo@mail.saitama-u.ac.jp
* Correspondence: colyercl@wfu.edu; Tel.: 336-758-4936

Contents

1. Materials and Methods

1-1. Reagents
1-2. Carbon dots

2. Results

Table S-1. Final Buffer Concentrations for Fluorimetry Samples.
Table S-2. Resolution Values for Fig. 4.
Figure S-1. Fluorescence spectra of apo- and holo-Tf without CDs and with Autoclave CDs.
Figure S-2. Representative electropherograms of apo-Tf, holo-Tf, and mixtures of apo- and holo-TF prepared in separation buffer or diluted separation buffer without CDs and with CDs.
Figure S-3. Introduction of CDs as separation adjuvants.
Figure S-4. Representative electropherograms of mixtures of apo- and holo-Tf with oven CDs synthesized from different precursors
Figure S-5. Full range of CDs concentrations tested with a mixture of apo- and holo-Tf.
Figure S-6. Separation buffer pH study.
Figure S-7. Tricine concentration effects.
Figure S-8. Tris concentration effects.
Figure S-9. Capillary inside diameter and temperature optimization.

1. Materials and Methods

1-1. Reagents

L-ascorbic acid (99%), gluconic acid (≥ 99%), and D (+) glucose (≥ 99%) were purchased from Sigma-Aldrich (St. Louis, Mo) and N-acetylneuraminic acid (≥ 98%) was purchased from Santa Cruz Biotechnology (Santa Cruz, CA).

1-2. Carbon Dots

In other syntheses, citric acid was replaced with other organic precursors, including ascorbic acid, gluconic acid, N-acetylneuraminic acid, or glucose, and CDs were prepared from these carbohydrate precursors by following the same procedure as for dry citric acid. All CDs were characterized by spectroscopic techniques (UV/Vis absorbance and fluorimetry) and by CE-LIF (Unpublished Results, L.Sirkisoon, 2014-2018 and T. Wittmann, 2016).

2. Results

Table S-1. Final Buffer Concentrations for Fluorimetry Samples

[Tf] μM	Final Buffer Concentration
0	50 mM tris - 200 mM tricine
0.5	49.9 mM tris - 199.6 mM tricine
5	49 mM tris - 196 mM tricine
10	48 mM tris - 192 mM tricine
25	45 mM tris - 180 mM tricine
50	40 mM tris - 160 mM tricine
100	30 mM tris - 120 mM tricine

Table S-2. Resolution Values for Figure 4.

Electropherogram	Forms of Tf	R_s
i	metallated and partially metallated	0.7
	partially metallated and demetallated	N/A
ii	metallated and partially metallated	1.3[a]
	partially metallated and demetallated	0.8[a]
iii	metallated and partially metallated	0.7
	partially metallated and demetallated	0.5
iv	metallated and partially metallated	0.8
	partially metallated and demetallated	0.5
v	metallated and partially metallated	0.8
	partially metallated and demetallated	0.8

[a]Resolution values differ from those in Figure 2 due to the fact that they were obtained for separations conducted on a different capillary and with a different buffer preparation

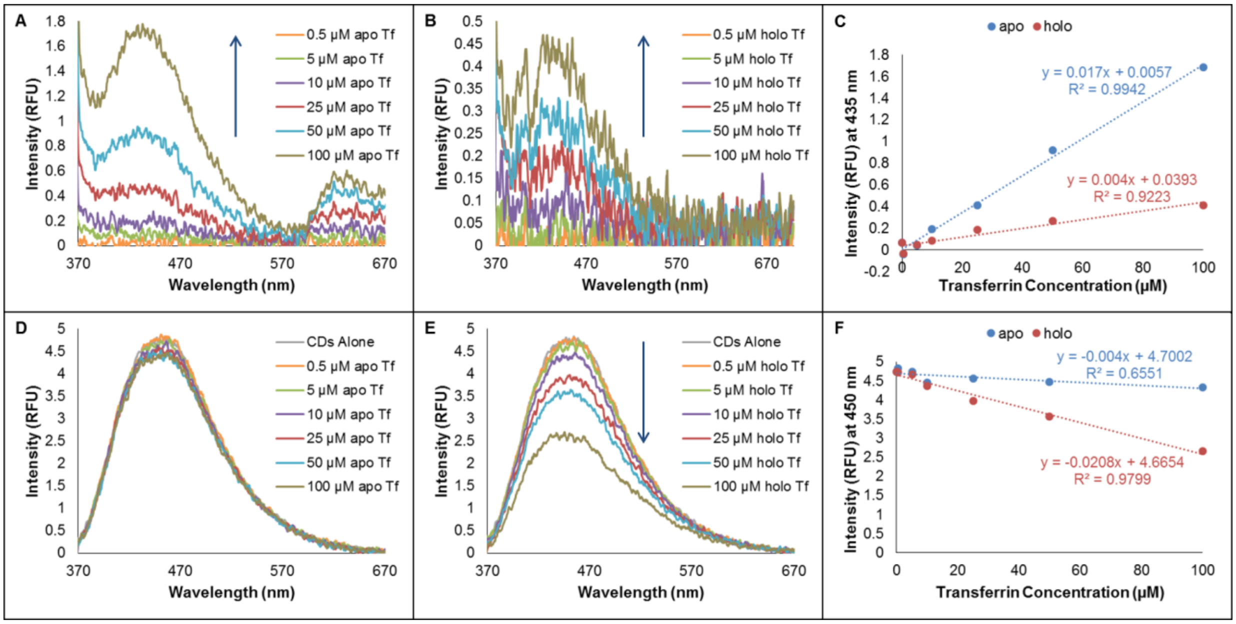

Figure S-1. Fluorescence emission spectra for samples of increasing concentrations (from 0.5 μM to 100 μM) of apo-Tf (**A**) and holo-Tf (**B**) and for samples of 35 μg/mL autoclave-synthesized citric acid CDs, with increasing concentrations (from 0.5 μM to 100 μM) of added apo-Tf (**D**) and holo-Tf (**E**). Fluorescence response in terms of intensity at the wavelength of maximum emission (435 nm for native Tf fluorescence (A and B), and 450 for CDs with Tf (D and E)) as a function of Tf concentration, for apo- and holo-Tf are shown in (**C** and **F,** respectively). All samples were prepared to the concentrations indicated in the Fig.s using 50 mM tris - 200 mM tricine (pH 7.4) buffer as diluent. The data were corrected for the respective native Tf fluorescence (from A and B) at each concentration. The excitation wavelength was 360 nm and the emission scan range was 365-700 nm.

An 8.5% quenching of fluorescence emission at 450 nm was observed for a 35 μg/mL autoclave synthesized CD sample upon increasing the concentration of apo-Tf from 0 to 100 μM (Figure S-1D). The fluorescence signal was quenched by as much as 43.7% upon the addition of up to 100 μM holo-Tf to the same CD sample (Figure S-1E). The intensities represented in Fig. S-1F were determined at the wavelength of maximum fluorescence emission (450 nm) after having corrected for native Tf fluorescence at each concentration (as shown in figures S-1 A and B) and applying a five-point boxcar smoothing. The extent of change in fluorescence of CDs as a function of Tf protein concentration is represented by the slopes of the response curves in Figure S-1F. The slope for apo-Tf is -0.004 RFU/μM indicating very little to no change in fluorescence of the CDs. However, the slope for holo-Tf is -0.0208 RFU/μM, revealing quenching of the CD signal with holo-Tf. However, this effect was not as dramatic as the quenching observed with the oven synthesized CDs.

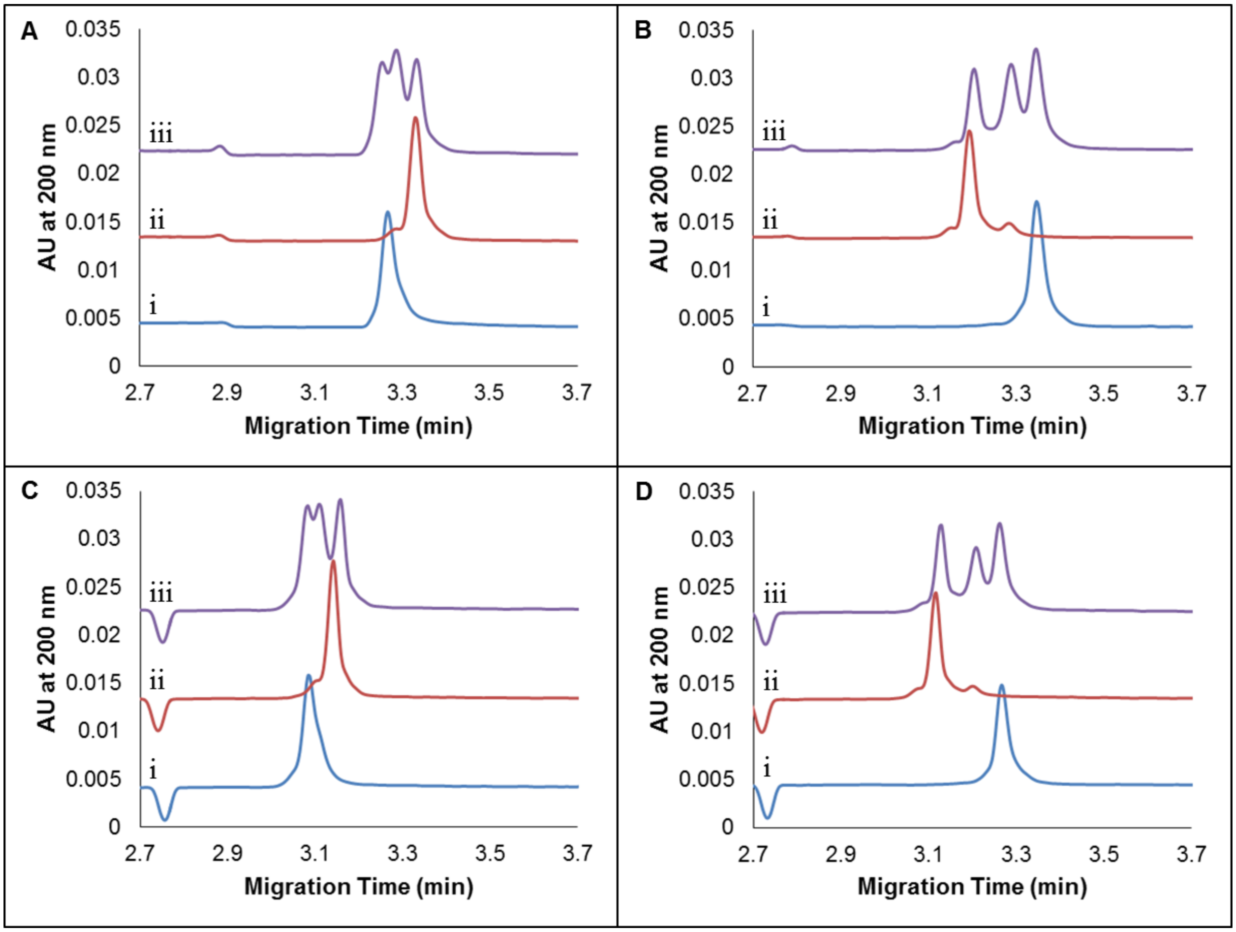

Figure S-2. Representative electropherograms for protein samples prepared in separation buffer or in diluted separation buffer without CDs (A and C, respectively) and with CDs (B and D, respectively) for: 25 μM apo- Tf alone (**i**), 25 μM holo- Tf alone (**ii**), and a mixture of apo- and holo-Tf (25 μM each, **iii**). Electropherograms are vertically offset for clarity.. A volume of 1.25 nL (5.2 sec at 1.3 psi) was injected and 20 kV was applied. The separation occurred on a Beckman Coulter P/ACE MDQ System coupled with a UV detector at 15°C on a 25 μm i.d. capillary with an effective length of 30 cm and a total length of 40 cm.

A broad signal with three discernable (but unresolved) peaks was observed for the mixture of apo- and holo-Tf without added CDs (Figure S-2Aiii), where the middle peak was the most intense. In the electropherogram with CDs present in the buffer system (Figure S-2Bii), the peaks were more resolved and their intensities were similar to one another. The peak area increased by 18.9% upon the addition of CDs as buffer additives.

An increase in migration time from 3.08 min without CDs to 3.27 min with CDs (a 5.9% increase in migration time, 8.2% decrease in peak height, and 12.4% decrease in peak area) was observed for apo-Tf (figures S-2Ci and Di, respectively). A slight shift from about 3.14 min without CDs to about 3.12 min with CDs (0.8% decrease in migration time, 23.1% decrease in peak height, and a 15.4% decrease in peak area) was observed for holo-Tf, along with an additional peak at about 3.20 min shown in figures S-2Cii and Dii, respectively. In the mixture of apo- and holo- Tf (figures S-2Ciii and Diii, respectively) without dots the middle peak was of equal intensity to the apo-Tf peak, while in the presence of CDs, the peaks are more resolved and the middle peak was the least intense and the combined area decreased by 13.1

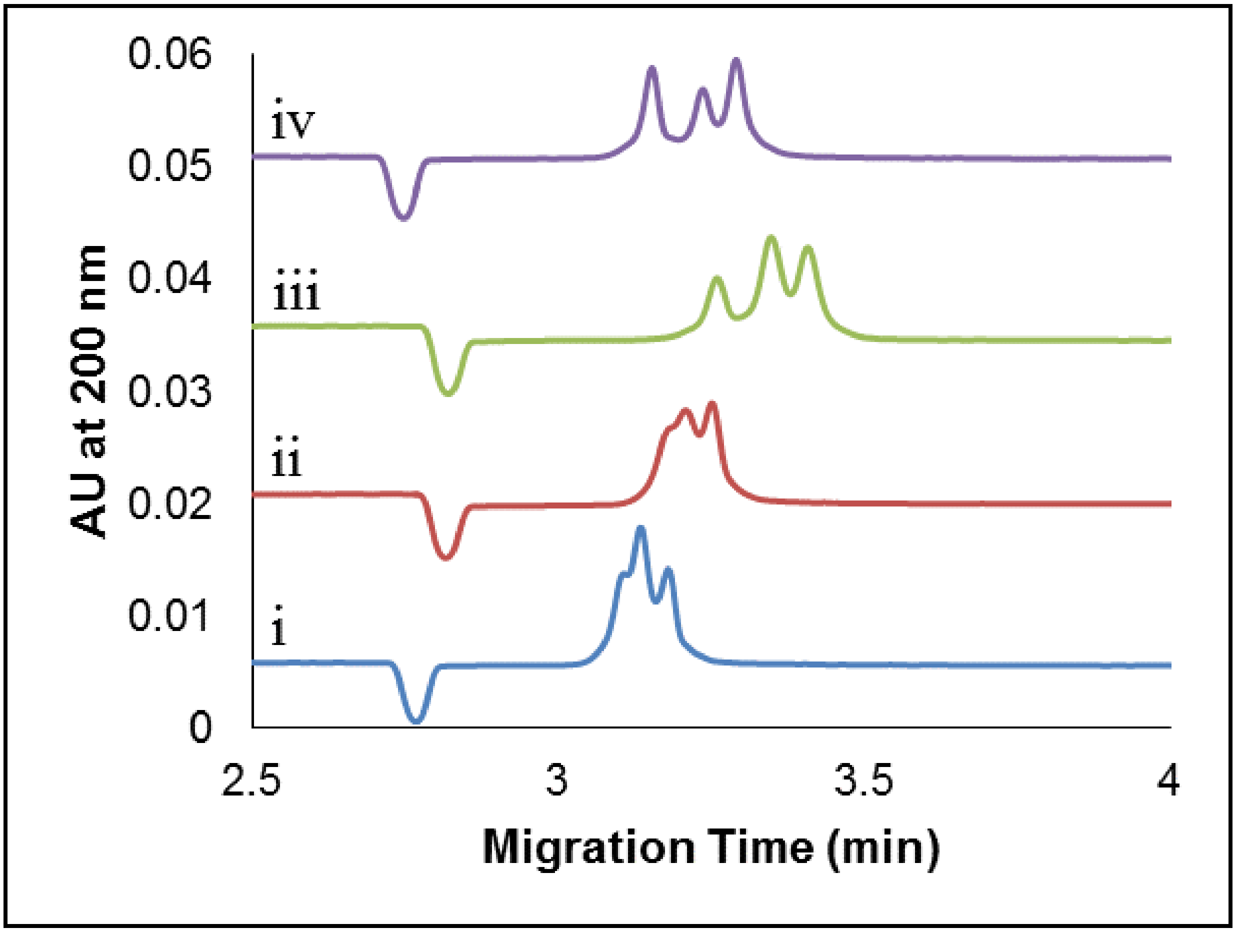

Figure S-3. Introduction of CDs as separation adjuvants for mixtures of apo- and holo-Tf (25 μM each) for no dots added (**i**), dots added only in the sample (**ii**), dots added only in the separation buffer (**iii**), and dots added to both the separation buffer and the sample (**iv**). Electropherograms are vertically offset for clarity. A volume of 1.25 nL (5.2 sec at 1.3 psi) was injected and 20 kV was applied. The separation occurred on a Beckman Coulter P/ACE MDQ System coupled with a UV detector at 15°C on a 25 μm i.d. capillary with an effective length of 30 cm and a total length of 40 cm.

In the absence of CDs (Fig. S-3a), a broad peak with three humps was observed with the middle peak as the most intense and the apo-Tf peak as a fronting shoulder. When CDs were introduced only to the sample (Figure S-3b) the migration of the broad peak shifted slightly later and still had three humps, but the middle and third hump (holo-Tf) were of equal intensity. In the case where CDs were added only to the separation buffer (Figure S-3c), the broad peak was shifted to a later migration time with more well defined peaks compared to the case without dots. Lastly, the dots were added to the separation buffer and to the sample (Figure S-3d), resulting in the first peak (holo-Tf) separating out from the second and third peak, and the middle peak was the least intense.

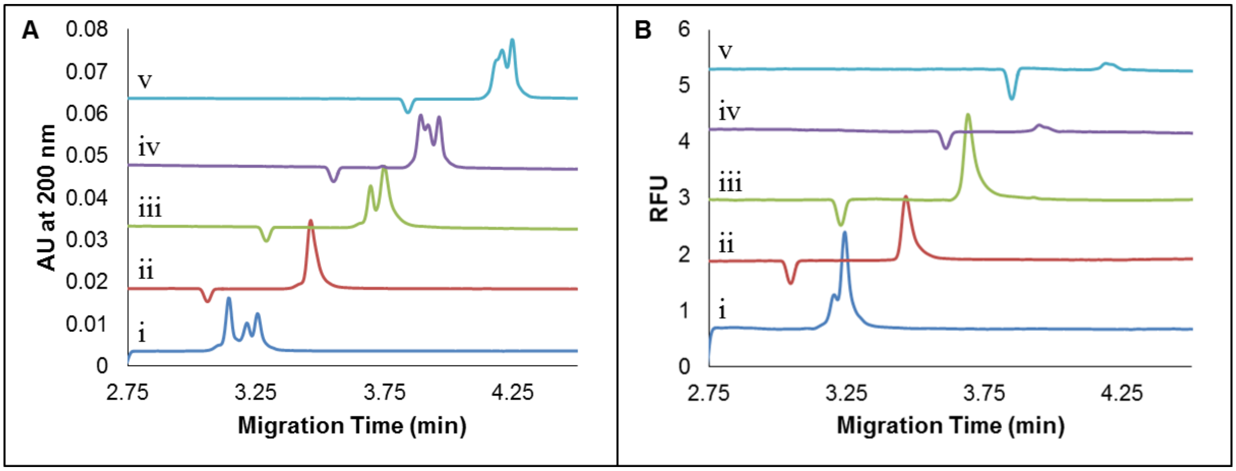

Figure S-4. Representative electrophregrams for mixtures of apo- and holo-Tf (25 μM each) with oven CDs synthesized from different precursors, analyzed by CE-UV/Vis (**A**) and CE-LIF (**B**). The precursors chosen were citric acid (**i**), ascorbic acid (**ii**), gluconic acid (**iii**), *N*-acetylneuraminic acid (**iv**), and glucose (**v**). Electrophereograms are vertically and horizontally offset for clarity. A volume of 1.25 nL (5.2 sec at 1.3 psi) was injected and 20 kV was applied. The separation occurred on a Beckman Coulter P/ACE MDQ System coupled with a UV detector for panel **A** and an LIF detector using a 375 nm laser with a 400 nm long pass filter for panel **B** at 15°C on a 25 μm i.d. capillary with an effective length of 30 cm and a total length of 40 cm.

CDs synthesized from citric acid gave rise to three peaks (Figure S-4Ai), CDs from ascorbic acid (Figure S-4Aii) and gluconic acid (Figure S-4Aiii) only gave rise to one or two peaks, and CDs from N-acetylneuraminic acid (Figure S-4Aiv) and glucose (Figure S-4Av) gave rise to peaks with shoulders that had the potential to have three peaks by CE-UV.

In CE-LIF studies, a single peak for the mixture of apo- and holo-Tf with CDs synthesized from citric acid, ascorbic acid, and gluconic acid was observed, and a tiny hump in the baseline for the Tf mixture with CDs from N-acetylneuraminic acid and glucose.

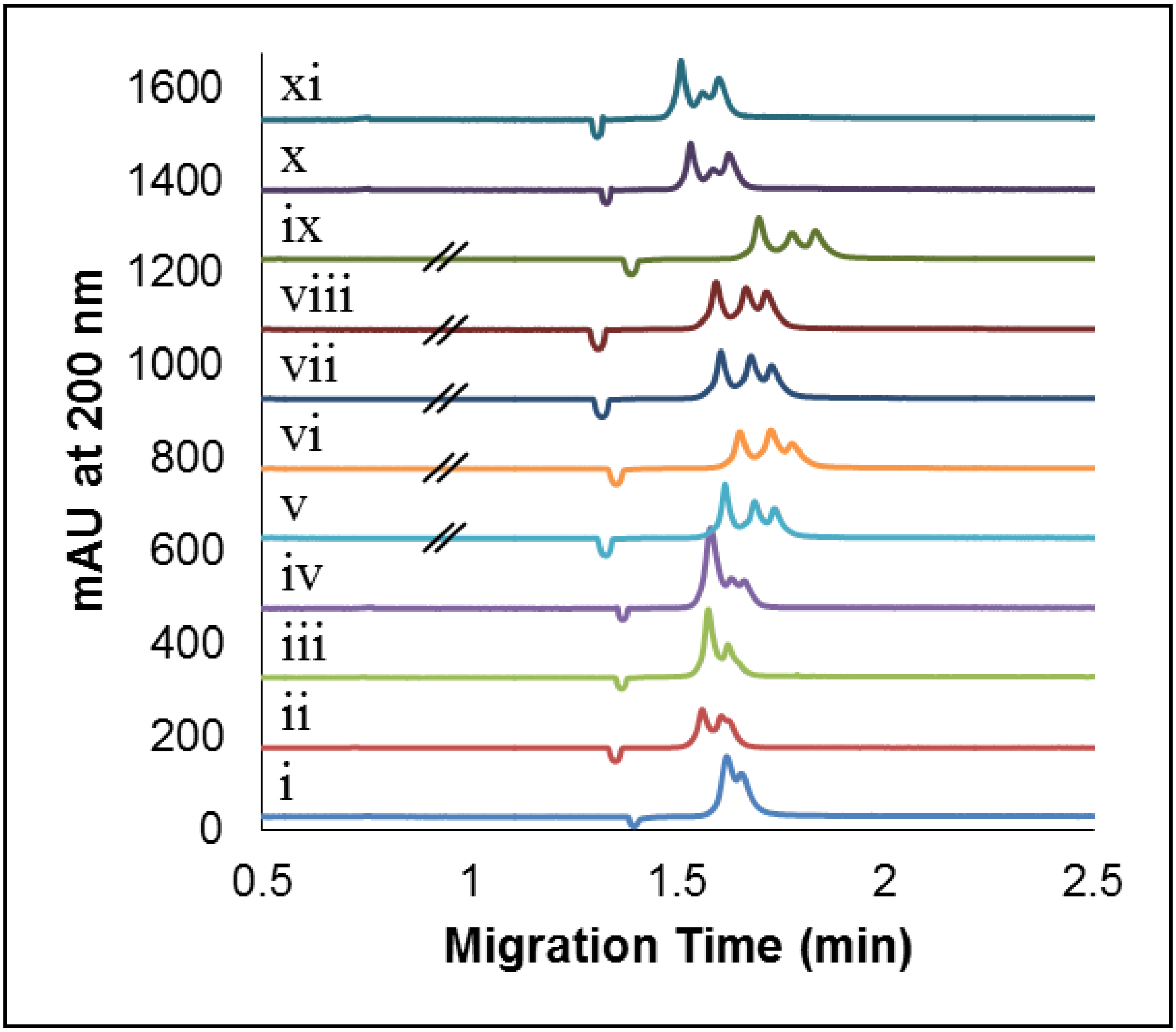

Figure S-5. Full range of CDs concentrations tested with a mixture of apo- and holo-Tf (25 μM each). Concentrations of CDs were (**i**) 2 μg/mL (**ii**) 5 μg/mL, (**iii**) 7 μg/mL, (**iv**) 10 μg/mL, (**v**) 25 μg/mL, (**vi**) 35 μg/mL, (**vii**) 50 μg/mL, (**viii**) 75 μg/mL, (**ix**) 100 μg/mL, (**x**) 250 μg/mL, and (**xi**) 500 μg/mL. Electropherograms are vertically offset for clarity. The electropherograms represented by (**v**) through (**ix**) were conducted on a different capillary and were normalized using an offset of 0.48 minutes. A volume of 5 nL (2.1 sec at 45 mbar) was injected and 20 kV was applied. The separation occurred on an Agilent G1600A CE with DAD UV/Vis Detector at 25°C on a 50 μm i.d. capillary with an effective length of 24 cm and a total length of 32.5 cm.

The samples in Figure 3 were prepared in 25 mM tris – 100 mM tricine buffer (pH 7.4), with a concentration of CDs in the sample equal to half of the reported concentration in the separation buffer. It should be noted that the electropherograms recorded with 35 and 100 μg/mL CDs (figures 3-iii and 3-iv, respectively and S-5.v - ix) were recorded using a different capillary and on a different day than those in figures 3-i, 3-ii, 3-v (and S-5. I - iv, x, and xi) and hence, the variability in their migration times can be attributed to expected differences in capillary wall charge, etc. To compensate for these differences, electropherograms 3-iii and 3-iv (and S-5. v - ix) are offset by 0.48 min relative to the other traces in Figure 3.

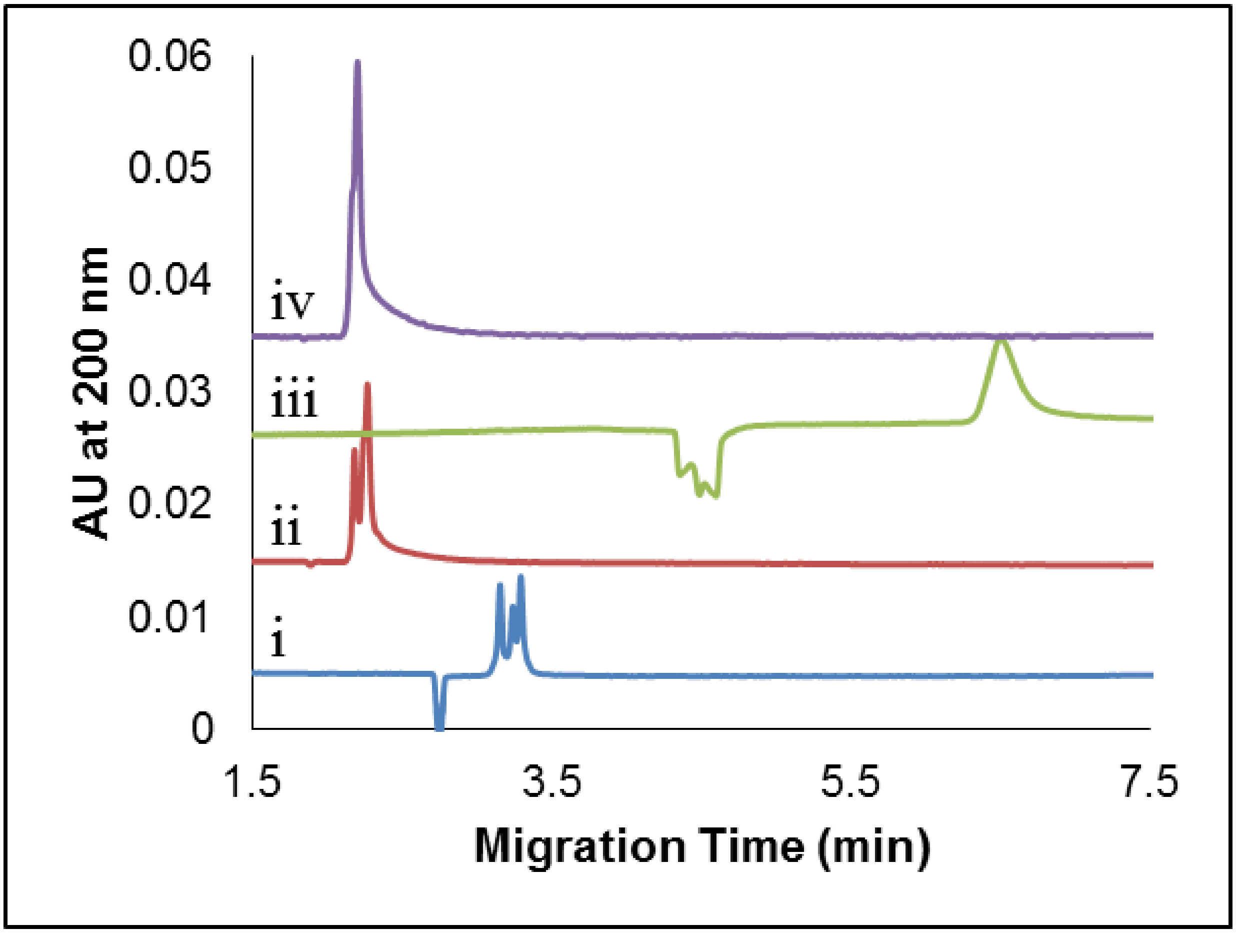

Figure S-6. Separation buffer pH study for mixtures of apo- and holo-Tf (25 µM each) with CDs in 50 mM tris – 200 mM tricine or 10 mM phosphate at pH 7.4 (**i** and **ii**, respectively) and pH 10.4 (**iii** and **iv** respectively). Electropherograms are vertically offset for clarity. A volume of 1.25 nL (5.2 sec at 1.3 psi) was injected and 20 kV was applied. The separation occurred on a Beckman Coulter P/ACE MDQ System coupled with a UV detector at 25°C on a 25 µm i.d. capillary with an effective length of 30 cm and a total length of 40 cm.

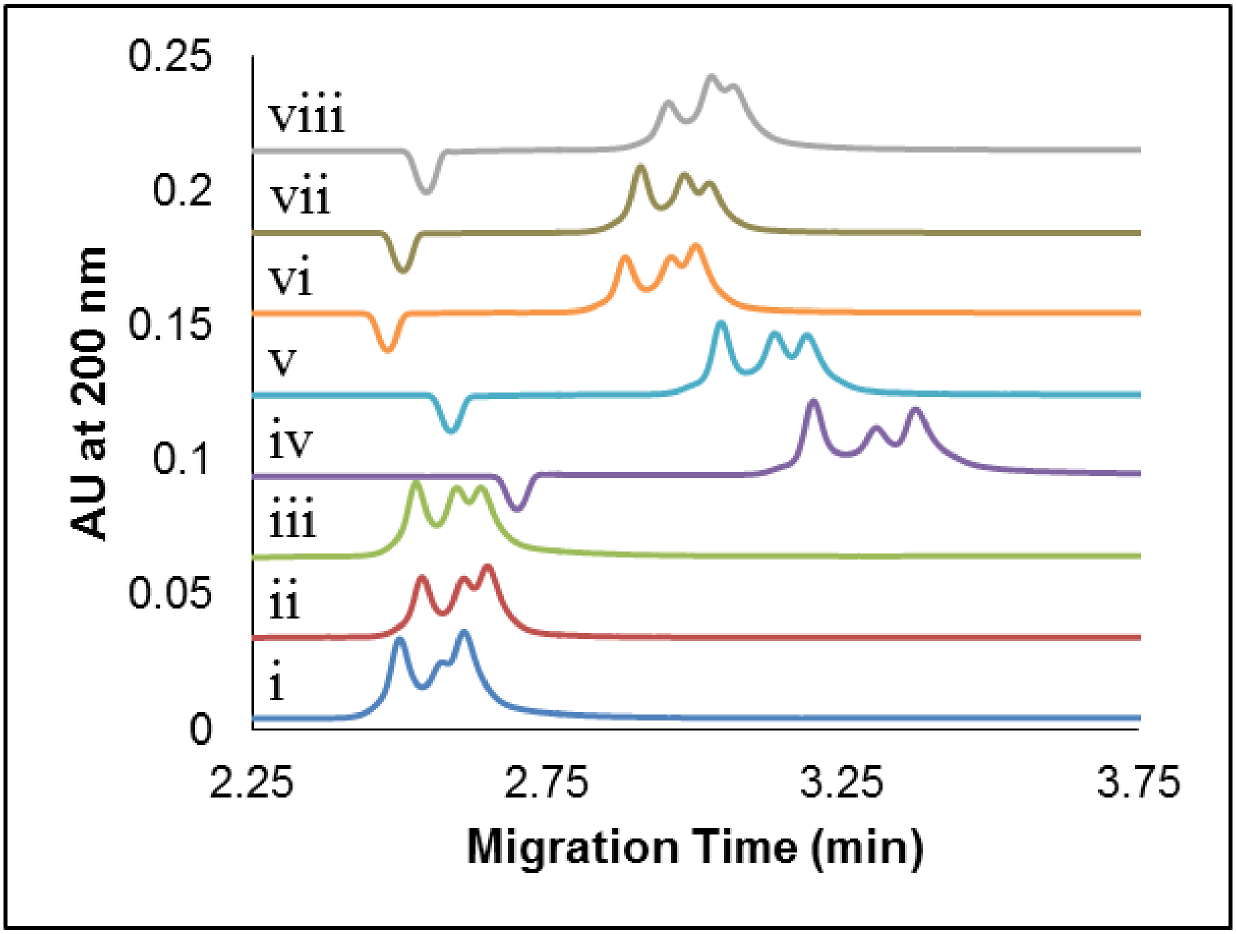

Figure S-7. Tricine concentration effects for mixtures of apo- and holo-Tf (25 µM each) with CDs at 100 mM (**i**), 150 mM (**ii**), 175 mM (**iii**), 200 mM (**iv**), 225 mM (**v**), 250 mM (**vi**), 275 mM (**vii**), and 300 mM(**viii**). Electropherograms are vertically offset for clarity. A volume of 1.25 nL (5.2 sec at 1.3 psi) was injected and 20 kV was applied. The separation occurred on a Beckman Coulter P/ACE MDQ System coupled with a UV detector at 25°C on a 25 µm i.d. capillary with an effective length of 30 cm and a total length of 40 cm.

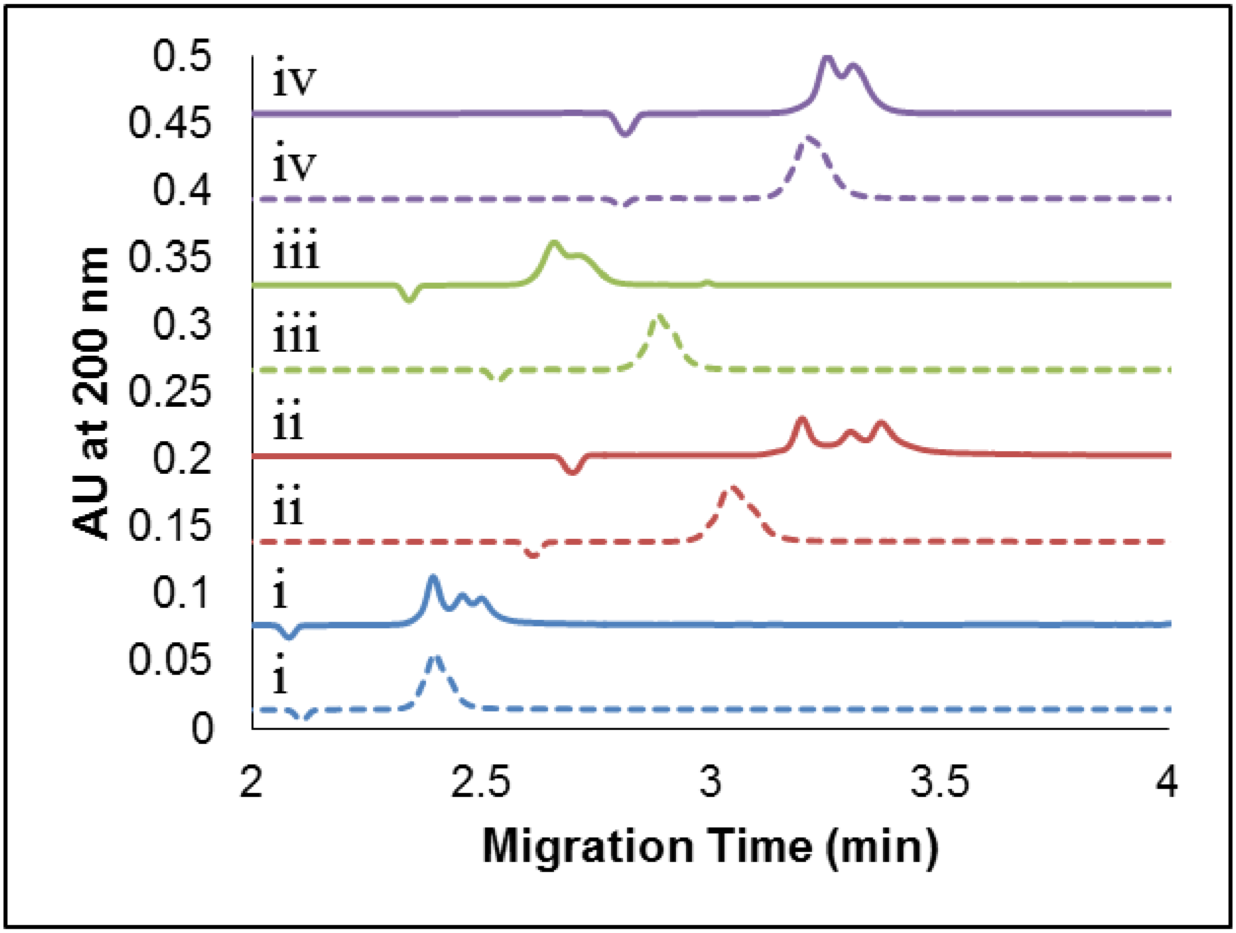

Figure S-8. Tris concentration effects for mixtures of apo- and holo-Tf (25 μM each) without CDs (dashed lines) and with CDs (solid lines) at 25 mM (**i**), 50 mM (**ii**), 75 mM (**iii**), and 100 mM (**iv**). Electropherograms are vertically offset for clarity. A volume of 1.25 nL (5.2 sec at 1.3 psi) was injected and 20 kV was applied. The separation occurred on a Beckman Coulter P/ACE MDQ System coupled with a UV detector at 25°C on a 25 μm i.d. capillary with an effective length of 30 cm and a total length of 40 cm.

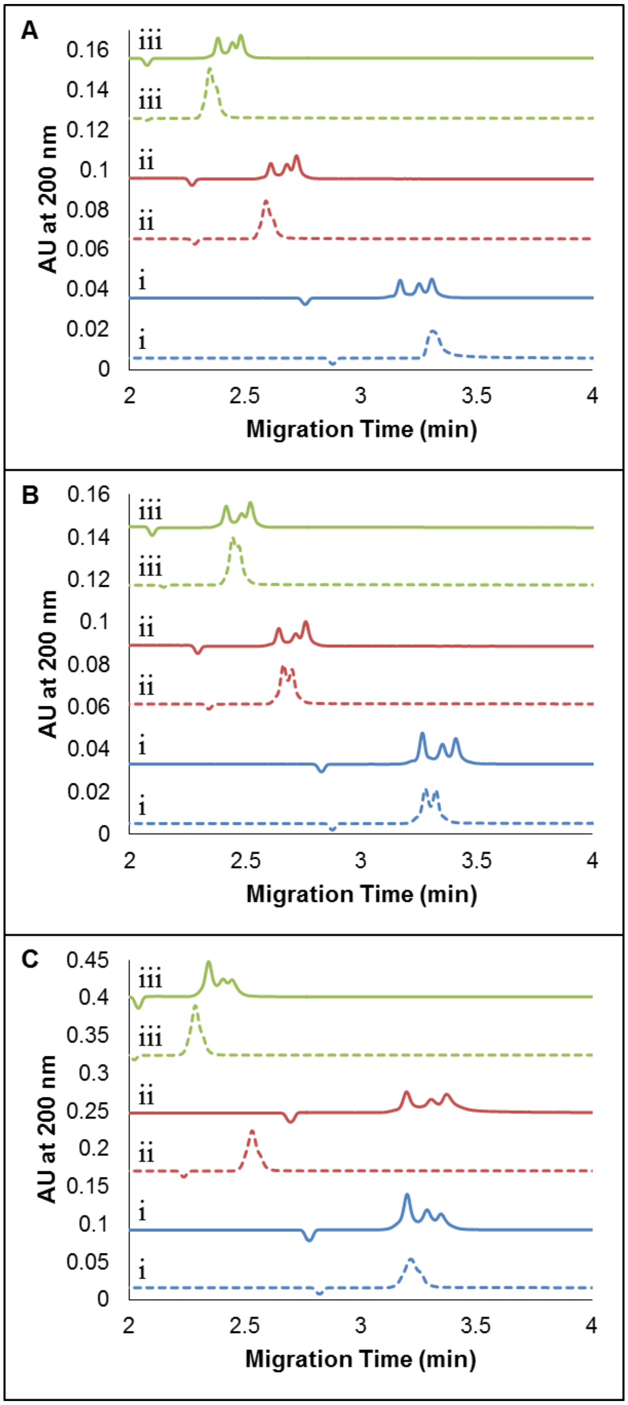

Figure S-9. Capillary inside diameter and temperature optimization for mixtures of apo- and holo-Tf (25 µM each) without CDs (dashed lines) and with CDs (solid lines), at 15°C (**i**), 25°C (**ii**), and 30°C (**iii**) on capillaries with inner diameters of (**A**) 20 µm, (**B**) 25 µm, and (**C**) 50 µm. Electropherograms are vertically offset for clarity. Volumes of of 0.8 nL, 1.25 nL and 5 nL were injected on the capillaries with inner diameters of 20 µm, 25 µm, and 50 µm, respectively, and 20 kV was applied. All capillaries were prepared with an effective length of 30 cm and a total length of 40 cm and installed on a Beckman Coulter P/ACE MDQ System coupled with a UV detector.
